# Anisotropic Porous Biodegradable Scaffolds for Musculoskeletal Tissue Engineering

**DOI:** 10.3390/ma2041674

**Published:** 2009-10-29

**Authors:** Eric L.W. de Mulder, Pieter Buma, Gerjon Hannink

**Affiliations:** Orthopaedic Research Laboratory, Nijmegen Centre for Molecular Life Sciences, Radboud University Nijmegen Medical Centre, PO Box 9101, 6500 HB Nijmegen, The Netherlands; E-Mails: e.demulder@orthop.umcn.nl (E.M.); p.buma@orthop.umcn.nl (P.B.)

**Keywords:** tissue engineering, scaffolds, anisotropy, solid free-form fabrication, musculoskeletal

## Abstract

It has been generally accepted that tissue engineered constructs should closely resemble the *in-vivo* mechanical and structural properties of the tissues they are intended to replace. However, most scaffolds produced so far were isotropic porous scaffolds with non-characterized mechanical properties, different from those of the native healthy tissue. Tissues that are formed into these scaffolds are initially formed in the isotropic porous structure and since most tissues have significant anisotropic extracellular matrix components and concomitant mechanical properties, the formed tissues have no structural and functional relationships with the native tissues. The complete regeneration of tissues requires a second differentiation step after resorption of the isotropic scaffold. It is doubtful if the required plasticity for this remains present in already final differentiated tissue. It would be much more efficacious if the newly formed tissues in the scaffold could differentiate directly into the anisotropic organization of the native tissues. Therefore, anisotropic scaffolds that enable such a direct differentiation might be extremely helpful to realize this goal. Up to now, anisotropic scaffolds have been fabricated using modified conventional techniques, solid free-form fabrication techniques, and a few alternative methods. In this review we present the current status and discuss the procedures that are currently being used for anisotropic scaffold fabrication.

## 1. Introduction

Damage to the tissues of the musculoskeletal system often result in failure to repair or the formation of regenerated tissue of inferior mechanical quality. Tissue engineering (TE) and regenerative medicine (RM) apply the principles of biology, embryology and engineering to develop functional substitutes for these damaged tissues. TE and RM typically involve implanting cells, with or without stimulating growth factors, into some form of supporting structural device, the so-called scaffold. The cells are allowed to grow, differentiate, and produce new tissue with their own specific extracellular matrix (ECM). In time, the formed tissue should also replace the space occupied by the scaffold. After resorption of the scaffold, the tissue formed should remodel into tissue with a large resemblance to the native tissue. This can be before or after implantation into the patient. In some cases the in-vitro culture period can be omitted and the scaffold can replace the deceased tissue directly, utilizing the host’s own body as a ‘bioreactor’. Cell harvest, scaffold seeding and implantation can then be performed in a single surgical event [1]. Taking this approach one step further, the scaffolding material can be inserted without cells. In this case, regeneration relies totally on the recruitment of native cells into the implanted scaffold and the subsequent deposition of an ECM [2]. Another option is the incorporation of cells directly into the scaffold during the scaffold fabrication process [3]. No matter which strategy is used, the scaffold itself is critical to the success of the construct, and in most cases actively directs the behavior of the cells within the scaffold.

Factors governing scaffold design are complex and include considerations of scaffold architecture, pore size and pore morphology, mechanical properties, surface properties, degradation speed, and degradation products. The scaffold architecture and surface properties should enhance initial cell attachment and allow efficacious seeding into the entire scaffold, or if this is difficult, migration into the scaffold. The scaffold should enhance the mass transfer of metabolites, provide sufficient space for remodeling of the newly formed tissue matrix and the development of vascularization. Furthermore, it must provide sufficient mechanical strength, particularly initially before the new tissue has matured into functional tissue. In other words, the scaffold degradation profile should be designed in a way it supports the construct during the remodeling process. Factors affecting the rate of remodeling include the type of tissue, and the anatomy and physiology of the host tissue.

A variety of materials have been used to produce scaffolds for the replacement and repair of damaged or traumatized musculoskeletal tissues. These materials include metals, ceramics, natural and synthetic polymers, and their combinations. In this review we will focus only on polymers. Both natural and synthetic polymers have been studied for use as scaffold materials in both TE and RM. Natural polymeric scaffolds have been produced from processed ECM constituents, such as collagen, elastin or hyaluronic acid. Natural polymeric scaffolds may also be produced from polymers derived from plants (alginate) or insects (chitosan). The most frequently used synthetic polymers are poly(lactic acid) (PLA), poly(glycolic acid) (PGA), poly(lactic-co-glycolic acid) (PLGA), poly(caprolactone) (PCL) and copolymers of these materials [4,5,6,7]. Generally, these materials have been approved for human use by the Food and Drug Administration. In addition, their degradation rates can be tailored to match that of new tissue formation. PLA is more hydrophobic and less crystalline than PGA and degrades at a slower rate, while PCL degrades even slower. The degradation rate of the polymers can be easily controlled by altering the ratio of the different copolymers in the formulation, by changing the chain-length of the monomers or by changing the cross-linking density [4,8]. In addition to degradation rate, certain physical characteristics of the scaffold must be considered when designing a substrate to be used in TE. In order to promote tissue growth, the scaffold must have a large surface area to allow cell attachment. This is usually done by creating highly porous scaffolds. In these foam-like scaffolds, the pores should be large enough and interconnected with each other to allow cells to penetrate deeply into the scaffold and to facilitate nutrient and waste exchange by cells deep inside the scaffold. Moreover, the scaffolds should have appropriate mechanical properties to provide temporary support within a specific application. These characteristics are often dependent on both polymer and method of scaffold fabrication.

Several methods have been developed to create highly porous scaffolds, including particle leaching [9,10], gas (CO_2_) foaming [11], freeze-drying [12], thermal induced phase separation (TIPS) [13,14], liquid/liquid phase separation in combination with freeze extraction [15], electrospinning [16], and particle sintering [17,18]. Also combinations of these techniques have been described [19]. More sophisticated tissue engineered constructs may utilize polymer scaffolds as a delivery vehicle for cells or bioactive proteins. Recently, a new class of scaffolds has been described in which synthetic polymers have been coated or blended with natural polymers [20,21].

Although these conventionally produced scaffolds hold great promise and have been applied to engineer a variety of tissues with varying success, most are limited by some flaws, which restrict their scope of applications [22]. In addition, the processing methods offer little capability to precisely control pore size, pore geometry, pore interconnectivity, spatial distribution of pores, and construction of internal channels within the scaffold.

The major limitation of the conventionally fabricated scaffolds is their isotropic nature. The tissue that is formed into the pores of an isotropic scaffold is a negative or mirror image of the scaffold itself, and since most tissue has significant anisotropic arranged extracellular matrix components and concomitant mechanical properties, the tissue formed has no structural and mechanical relationship with the native tissue [23]. For complete regeneration of the tissue a second differentiation step is needed during and after resorption of the isotropic scaffold. It is doubtful if such a plasticity can be expected from already final differentiated tissue. It would be much more efficacious if the newly formed tissues in the scaffold could differentiate directly into the anisotropic organization of native tissues. Anisotropic scaffolds with a porous, tubular or other structure, that enable direct differentiation into the native tissue configuration might be extremely helpful to realize this goal.

The introduction of solid free-form (SFF) technologies have initiated the start of a new exiting and revolutionary era for scaffold design and production [22]. SFF techniques are computerized fabrication techniques that can produce highly complex, layer-by-layer build, three-dimensional physical objects, using data generated by computer aided design (CAD) systems or computer-based medical imaging modalities. The most promising techniques to generate scaffolds with a highly anisotropic pore structure are SFF technologies, such as 3D fiber deposition (3DF), fused deposition modeling (FDM), bioplotting, stereolithography (STL), and selective laser sintering (SLS), but also modified “conventional” techniques, such as modified thermal induced phase separation (modified TIPS) and electrospinning. In this review we will present the current status and discuss the procedures which are currently in use for anisotropic scaffold production.

## 2. Solid Free-Form Fabrication

Solid free-form technologies, also commonly known as rapid prototyping (RP), are computer aided manufacturing (CAM) techniques that can rapidly produce highly complex three dimensional physical objects using data generated by computer aided design (CAD) programs, or converted from computer-based medical imaging modalities, such as MRI and CT [24,25]. Unlike conventional computerized machining processes, which involve the removal of materials from a stock, SFF techniques use the underlying concept of layered manufacturing whereby three-dimensional objects are fabricated with layer-by-layer building via the processing of solid sheet, liquid or powder material stocks [22]. The flexibility and outstanding manufacturing capabilities of SFF have been employed for biomedical applications, such as production of scale replicas of human bones [26], body organs [27], and advanced customized drug delivery devices [28]. The direct utilization of CAD models as inputs for scaffold fabrication allows complex scaffold designs to be realized. Patient specific data and scaffold structural properties required for regenerating specific tissues can be incorporated into scaffold design via CAD. The application of CAD strategies in conjunction with SFF fabrication will allow scaffolds with highly uniform pore morphologies, unlimited range of pore sizes, porosities and complete pore interconnectivity to be realized with unprecedented accuracy and consistency for patient specific application [22]. In addition, SFF techniques employ a diverse range of processing conditions which include solvent and/or porogen free processes and processing at room temperature. Some SFF techniques allow pharmaceutical and biological agents to be incorporated into the scaffold during fabrication [28,29,30]. The utilization of CAD will also allow scaffolds with optimized mechanical and structural properties to be fabricated. The following sections will focus on SFF techniques that have been used for fabricating anisotropic scaffolds.

### 2.1. 3D Fiber Deposition/Fused Deposition Modeling

**Figure 1:**
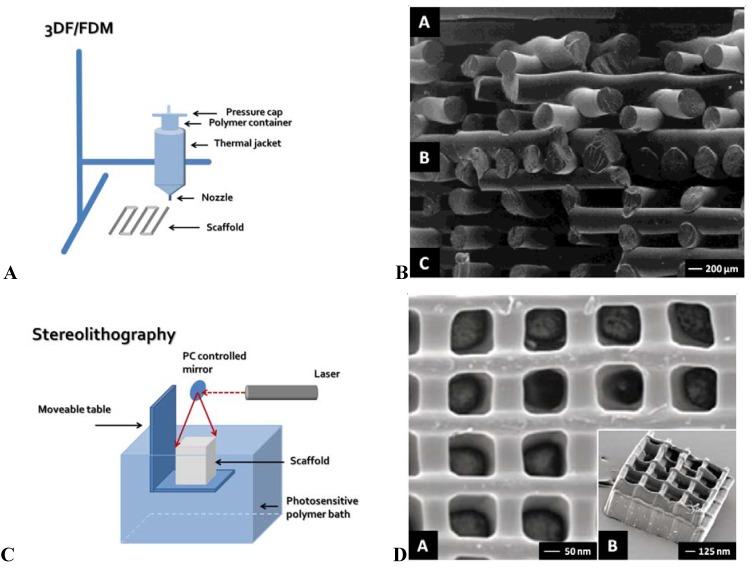

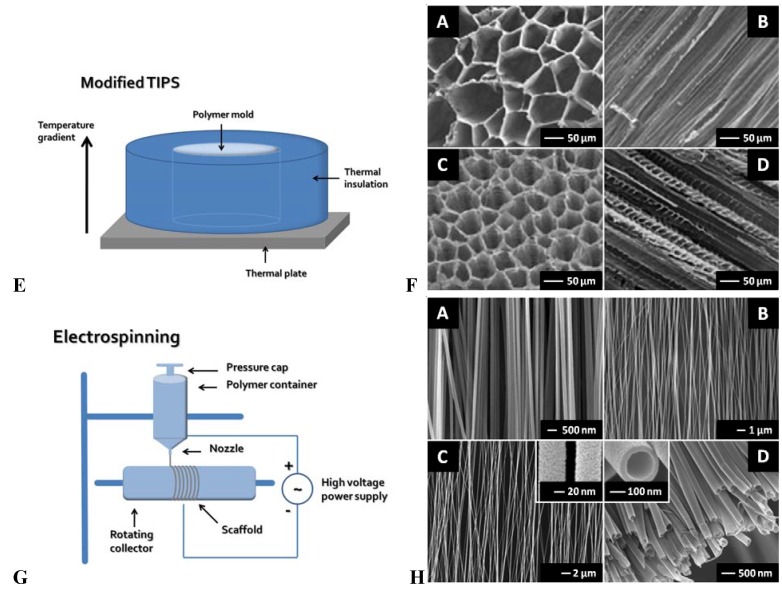
**(A)** Schematic representation of 3D fiber deposition (3DF)/Fused deposition modeling (FDM). **(B)** SEM micrographs of a PCL scaffold with varying multiple-layer design prepared using 3DF/FDM, showing a scaffold architecture with (A) a 0/60/120° lay-down pattern for the top part, (B) a nonporous structure for the middle part, and (C) a 0/90° lay-down pattern of the bottom part of the scaffold. Reproduced and modified with permission from Hutmacher *et al*. [99]. Copyright John Wiley & Sons, Inc. **(C)** Schematic representation of stereolithography (STL). **(D)** SEM micrographs of a STL prepared scaffold, showing (A) a single-layer scaffold, and (B) a multilayered scaffold. Reproduced and modified with permission from Mapili *et al*. [59]. Copyright John Wiley & Sons, Inc. **(E)** Schematic representation of modified thermal induced phased separation (TIPS). **(F)** SEM micrographs of the uniaxial microtubular scaffolds prepared using modified TIPS, showing (A) a cross-section of a scaffold prepared from 2.5% (w/v) polymer/benzene under liquid nitrogen, (B) a longitudinal section of this scaffold, (C) a cross-section of a scaffold prepared from 3.0% (w/v) polymer/benzene under liquid nitrogen, and (D) a longitudinal section of this scaffold. Reproduced and modified with permission from Chen *et al*. [72]. Copyright John Wiley & Sons, Inc. **(G)** Schematic representation of modified electrospinning. **(H)** SEM micrographs of scaffolds prepared with electrospinning, showing (A-C) aligned nanofibers, and (D) uniaxially aligned nanotubes. Reproduced and modified with permission from Li *et al*. [100]. Copyright Wiley-VCH Verlag GmbH & Co. KGaA.

3D fiber deposition and FDM are similar techniques. The majority of 3DF/FDM techniques are nozzle-based systems. Polymers are thermally processed when they pass a nozzle and are deposited as a relatively thick fiber on a collector plate. After deposition on the collector plate, the fibers cool down, solidify and the scaffold is ready for use (Figure 1AB). Fiber diameter can be controlled by changing parameters, such as nozzle diameter, deposition speed, extrusion speed and viscosity of the polymer, and can range between 170-750 µm [31,32,33]. A wide range of polymers, including hydrogels, have been used in 3DF/FDM [34,35,36,37]. However, most of the produced scaffolds have an internal isotropic organization. Each successive layer is deposited under a different angle, creating a specific lay-down pattern [38,39]. Most used lay-down patterns are 0/90º, 0/45/90/135º and 0/60/120º. The compressive stiffness of the scaffold can range from 4–80 MPa, depending on the lay-down pattern, fiber thickness, and the polymers used for fabrication [38]. Using a 0/45º lay-down pattern, the compressive stiffness in the z plane is different compared to the compressive stiffness in the x/y plane, thereby resulting in mechanical anisotropy. Anatomically 3D fiber deposited trachea, menisci and vertebra have recently been fabricated from patient-derived computer-based medical imaging modalities [25,35,40]. In addition, shell/core fibers have been produced by exploiting viscous encapsulation, a rheological phenomenon in polymeric blends flowing through narrow ducts [41]. Furthermore, by printing fibers directly aligned in one layer followed by a semi-open layer, anisotropic scaffolds with aligned channels can be created (unpublished data). In this way, also nutrient supply in the deeper zones of the scaffolds could be facilitated [42].

### 2.2. Bioplotter

Bioplotting was first described by Wilson and Boland [43]. They modified a commercial inkjet printer into a custom-made bioplotter. After cleaning the ink cartridges, the containers were refilled with solutions containing marker proteins (y-biotine, steptavidine, streptavidine-BSA and BSA) or with endothelial or smooth muscle cells. The resolution of this technique has been shown to be approximately 25-50 µm in both the x and y directions [43,44]. Using this technique, complex structures containing viable cells after printing have been produced [45]. However, after printing a 25% cell death was observed [43]. It has been shown that cell death could be strongly reduced by avoiding dehydration of the cells after printing by increasing the water content of the gels in which the cells are encapsulated [43,46,47]. Incorporation of growth factors or other biological cues, such as calcium phosphates, specific adhesive peptides, and collagen can modulate cell function and degradation of the scaffold [34]. In this way, an instructive microenvironment for embedded or seeded cells can be created [48]. Cohen *et al.* were able to fabricate a geometrically complex structure in the shape of a meniscus using alginate as scaffold material [44]. However, so far, no anisotropic scaffolds have been fabricated using bioplotting. Using multiple cartridges, zonal distribution of multiple cell types and biological cues anisotropic scaffolds should be relatively easily achieved [34].

### 2.3. Stereolithography

Stereolithography is the oldest RP technique [49]. It is based on spatially controlled photopolymerization of a liquid resin by a laser. As the resin only solidifies where illuminated, a specific pattern can be created in one single layer. By repeating this process, three-dimensional structures can be built in a layer-by-layer manner (Figure 1CD) [50,51]. In conventional STL machines one laser beam is used for photopolymerization. However, recently new systems have been developed which use two lasers in the near infrared spectrum, the so-called two photon polymerization technique (2PP) [52,53,54,55].

A prerequisite for polymers to be used in STL are end-groups that can be polymerized by light [3,50,54,55,56,57,58,59,60]. Epoxy resins are very popular for the generation of non-resorbable STL molds. These molds are used to shape polymers into conventional made scaffolds for various tissue engineering or regenerative medicine purposes [61,62,63]. Two resorbable materials are particularly interesting for STL scaffold production. First, scaffolds based on poly-DL-lactic acid (PDLLA) are very popular for bone tissue engineering. A second very promising material that has been recently introduced is poly(propylene fumarate) (PPF) [64,65]. Hydrolysis of the ester bonds allows PPF to degrade into fumaric acid and propylene glycol, both of which are non-toxic products [55]. Scaffolds made from PFF have mechanical properties in the MPa range (15-40 MPa) and are also suitable for bone tissue engineering.

The accuracy of STL is dependent on a number of parameters. The most important ones are the machine, the method (one photon versus two photon polymerization) [49,53,54], the laser power [53,65], the scan speed [65], and the length of the laser beam path into the polymer [66]. In general, commercial STL machines have a resolution in the order of 200–250 µm. Some claim a resolution of 75 µm or even lower but then they might fail to create open pores [59]. Others claim even accuracies of 10–100 µm, however this has been shown to be the ultimate limit of accuracy with conventional machines [54]. The penetration depth, and by that the accuracy in the z-direction, may be increased by adding specific dyes to the polymer which reduce the penetration depth of the UV beam. With conventional STL the layer thickness can then be reduced from 29 to 7 µm [66]. The structural resolution of 2PP polymerization is in general much higher and can be even lower than 100 nm [53,54].

A matter of attention is the matching between CAD files and the produced scaffold structures. Shrinkage of resins used for STL is a general phenomenon and might be up to 25% [64,65]. However, if the shrinkage is isotropic, which is generally the case, the CAD files might be adapted to the expected percentage of shrinkage of a specific polymer with a good matching as a result [3,65]. Generally, the difference between the CAD file and the produced structure will not exceed 0.5%. For porous scaffolds the differences between CAD files and the produced pores have been described to be larger, ranging from 0.2% for the large pores to 8.3% for the very small pores [65].

Amongst the pitfalls in STL are diluting the polymers before the polymerization process and the use of non-reacted chemicals after polymerization [66]. Diluting the polymers negatively influences the mechanical properties of the produced structures [67]. Non-reacted chemicals inside the scaffold can potentially cause problems if they are not adequately removed from the fabricated structures. Particularly non-reacted photo-initiator might be cytotoxic [68]. The use of biocompatible non-reacted polymers and non-reactive diluents would be a solution [66].

Functional studies on STL scaffolds are scarce. Engelmayer *et al.* have shown that pore dimensions influence collagen orientation and deposition by rat skin fibroblasts [69]. Using 2PP STL, sub-micron needle like structures have been produced on which the reaction of fibroblast cells (NIH-3T3) and epithelial cells (MDCK) has been shown to be dependent on the pattern of actin microfilament bundles in the cells. For bone TE scaffolds with channels were produced to study the optimal channel diameter to facilitate cell ingrowth [70]. Functional animal studies related to the in-vivo tissue reactions are very scarce. The group of Jansen studied the soft and hard tissue response to photo-crosslinked PPF scaffolds in a rabbit model [58]. These scaffolds appeared to induce a mild inflammatory reaction in both tissue types. A powerful future application might be the blending of cells into SLT scaffolds during photopolymerization [56].

## 3. Modified Conventional Techniques

Conventional methods for manufacturing scaffolds include particle leaching [9,10], gas (CO_2_) foaming [11], freeze-drying [12], thermal induced phase separation (TIPS) [13,14], liquid/liquid phase separation in combination with freeze extraction [15], electrospinning [16], and particle sintering [17,18]. However, there are inherent limitations in these processing methods. These methods offer little capability to control pore size, pore geometry, pore interconnectivity, spatial distribution of pores and construction of internal channels within the scaffold. [71]. Consequently, it has been tried to modify the conventional techniques to overcome these inherent process limitations [17,72,73,74,75]. The following sections will focus on modified conventional techniques that have been used to fabricate anisotropic scaffolds.

### 3.1. Modified Thermal Induced Phase Separation (TIPS)

A commonly used procedure to create porous polymer scaffolds is TIPS. This technique is based on the principle that a homogeneous polymer solution made at elevated temperature is converted via the removal of thermal energy (cooling) into two-phase separated domains. Through extraction, evaporation or sublimation the solvent containing phase will be removed and give rise to the pores of the scaffold. Two different phase separations have been described, namely solid-liquid and liquid-liquid phase separation. In solid-liquid phase separation the temperature is lowered to solidify the polymer solution. The solvent will form crystals and thereby separate the polymer from the solvent. Finally, the crystals are removed which exposes the pores. In the second method, liquid-liquid phase separation, phase separation is based on a polymer-rich and a polymer-lean phase. The solvent part will be present in the polymer lean phase which will be processed further to create the pores in the scaffold [14,76]. For example, polymers such as PLA and PLGA are diluted in dioxane/water in the desired polymer concentration and subsequently cooling down of the polymer induces the phase separation. A porous foam-like scaffold is available after removal of the solvent in a freeze-dryer. The formed scaffolds have a randomly distributed isotropic pore structure [77,78,79].

In the modified TIPS a uni-axial thermal gradient is applied between top and bottom instead of uniformly cooling during the phase separation step (Figure 1EF) [80]. Via insulation of the walls of the polymer/solvent container a longitudinal temperature gradient is created resulting in longitudinal crystal formation. The result is a scaffold containing micro-channels instead of pores, which are orientated in a top to bottom direction. The diameter of these micro-channels can range between 15–240 µm [72,81,82]. The diameter can be controlled by changing the thermal gradient and/or the proportion polymer/solvent. Using a higher thermal gradient and higher polymer concentration the diameter of the channels has been shown to decrease. The micro-architecture of the channels can be influenced by the type of solvent used. Using benzene as a solvent, microtubules will be open, while with dioxane the channels have ladder-like structures [72,80]. The anisotropic structural properties of the scaffold is reflected in higher compressive modulus and yield strength in the longitudinal direction compared to the transverse direction [72,80]. Culture studies show that cells grow along the direction of the microtubules and synthesize oriented neo-tissue [83]. Modified TIPS has also been applied to natural polymers such as collagen. A temperature gradient between bottom and top of the container, according to the Bridgman technique [84], resulted in an ice front growth in an opposite direction than the temperature gradient [85]. Applying this temperature gradient to collagen suspensions resulted in unidirectional collagen scaffolds, also with some ladder-like characteristics [86,87,88]. The channels were in the range 20-40 µm and were predominantly controlled by the temperature gradient and solvent type (either ethanol or acetic acid). The scaffolds can be molded in discs or tube like shapes. It should be noticed that after polymerization skin formation can occur on the edge, thereby limiting cell penetration into the scaffold.

### 3.2. Electrospinning

A conventional method for fiber deposition is electrospinning. Using a high voltage/low current a very thin fiber (tenths of nanometers) can be produced from a polymer droplet, which is directed toward a grounded counter plate electrode, the so-called collector plate. On a static collector plate the fibers will be displayed in a random orientation. Scaffolds can be fabricated in various different tubular configurations with different surface patterning using different shaped, static 3D, columnar patterned collectors [89]. With help of a rotating cylinder or rotating disc type collector it is possible to produce orientated/aligned fibers (Figure 1GH) [90,91]. It has been shown that fiber diameter and degree of fiber alignment can determine the behavior of cells [16]. Cells orientate themselves and migrate into the direction of the fiber orientation with higher proliferation and synthetic rates [92,93]. Aligned nanofibers seeded with ligament fibroblasts induced more elongated fibroblasts compared to randomly orientated fibers, and these cells also produced more collagen compared to cells on non-aligned scaffolds [94]. Multiple spinneret tips with different polymers can create multilayer scaffolds [95]. These layers can also be separated by seeded cells. Using different cell types in between layers, complex 3D structures with living cells have been produced [96]. With a set-up using a syringe-inside-a-syringe core/shell nanofibers have been created [97]. This technique is has also been used to produce aligned collagen nanofiber scaffolds with a similar cellular response to fiber orientation as described above [98].

## 4. Alternative Methods to Create Anisotropic Scaffolds

Using the techniques outlined above, the production of aligned channels in a scaffold has been realized. However, most of the above mentioned techniques are technically demanding. Alternative methods might be cheaper, more easy to use, or useful if the size of features in the scaffolds is limited by the resolution of the technique. Therefore, a number of alternative methods have been proposed to incorporate aligned channels into the general structure of the scaffold. Some of these alternative methods can be applied directly during scaffold polymerization, while others are applied after polymerization as a post-processing step.

### 4.1. Direct Methods

Most polymers are in the liquid phase before molding. Using arrays of needles and/or wires placed in the mold, porous scaffolds with aligned channels can easily be fabricated. The diameter of these channels depends on the thickness of the needles and/or wires used. The architecture can vary on the needle array applied [101,102]. Unfortunately, removal of the needles and/or wires after polymerization has been shown to damage these scaffolds. To prevent damage, coated wires and/or needles have been used [103]. After polymerization of the scaffold, the coating on the wires or needles can be dissolved, facilitating the removal of the wires and/or needles. An alternative approach is to use wires that can dissolve completely [18,103]. Nazhat *et al.* incorporated unidirectional aligned soluble 30–40 µm diameter phosphate glass fibers (PGFs) into dense compacted collagen scaffolds [104]. The degradation time of PGFs can range from minutes to years depending on their chemistry. Their biocompatibility has been demonstrated with a number of cell types [105,106]. In addition, the use of phosphate glass particles as reinforcing agents, and eventually as porogens through their degradation in synthetic biodegradable composites, has been carried out in PCL [107,108] and lactide [109,110] matrices developed for drug delivery and tissue engineering. Therefore, PGFs might also be used as channel-creating devices in synthetic biodegradable composites. Besides solvable wires or retracting wires, it is also possible to leave the wires inside the scaffold but then the effect of creating access for mass transport is lost [111].

Another direct method to create anisotropic scaffolds is the use of conventional porogens, such as sugar, salt, alginate, or bovine serum albumin micro-bubbles [112,113,114,115]. Capes *et al.* have managed to arrange sugar strands in a mold [116]. Subsequently, the polymer solution was casted over the sugar strands, and after polymerization the strands were washed out. Similar methods can be applied using other porogens like paraffin, sodium alginate and/or gelatine [117].

### 4.2. Post-Processing Methods

Various methods have been described to create channels in scaffolds after polymerization. Silva *et al.* produced an array of aligned 400 µm thick channels into a random, porous PDLLA scaffold structures using a computer controlled drill system [102]. Using relatively thin polymer sheet, excimer laser ablation has been used to create specifically sized channels in polymer scaffolds [118,119,120,121]. For musculoskeletal tissue engineering purposes stacking these single-layer sheets might be an option. Recently, Vishnubhatla *et al.* have fabricated micro-channels in fused silica by femto-second laser irradiation [122]. They have created cylindrical micro-channels with uniform cross-sections and a length of 4 mm. As mentioned above, Capes *et al.* used a traditional solid porogen technique for the fabrication of PGLA scaffolds [116]. Besides the use of close-packed sugar strands to create a 3D polymer scaffold with multiple aligned channels, they also created single-layer scaffolds using sugar spheres. Multi-layer scaffolds were created by stacking the single-layer to form a 3D structure. Also known porogens such as, paraffin, sodium alginate, and gelatine might be used to create anisotropic scaffolds using single-layer stacking. Using phase separation micromolding, Papenburg *et al.* reported a one-step method to fabricate highly porous micro-patterned polymer 2D scaffold sheets [123]. These 2-D micropatterned sheets can be built into a 3-D scaffold by multi-layer stacking immediately after casting. Residues of the solvent still present in the sheets enabling the layers to bond. Stacking was achieved through two different methods: by either clamping several films between glass-plates or rolling up one or more sheets around a tube. By tuning the size of the channels and the sheet thickness, the scaffold architecture can be designed.

## 5. Discussion

Tissue engineered biomaterials should ideally bear close resemblance to the *in vivo* mechanical and structural properties of the tissues that they are intended to replace. Since mechanical loads can vary spatially and temporally within the tissues of an organ, they exhibit complex, mechanically anisotropic behaviors optimized for their respective physiological functions [124].

Many tissues in the musculoskeletal system, particularly those that bear mechanical loads in a defined direction, exhibit preferential fiber alignment [125]. This alignment endows such tissues to functional properties that vary depending on the testing direction. For example, in tendons and ligaments, tensile properties are 200-500 times higher along, compared to perpendicular to, the fiber direction [126]. In articular cartilage, tensile properties are greatest in the superficial zone along the split line direction [127,128]. In the meniscus, circumferentially oriented collagen fibers predominate [129], resulting in higher tensile properties in the circumferential compared to the radial direction [130,131,132]. When damage occurs, the architecture is interrupted and the ability of the tissue to withstand load is compromised. As such, the architecture must be one of the first considerations when engineering replacement constructs [133].

Using conventional scaffolds, it has been shown that the tissues formed had no relationship with the native tissue but were oriented into the direction of the isotropic distributed pores [23]. Differentiation of the newly formed tissue directly into the anisotropic organization of the native tissue would be much more efficacious. Therefore, one of the considerable challenges remaining in the field of tissue engineering is to produce a construct with an anatomically correct architectural framework, both in terms of cell morphology and matrix deposition [125].

The use of SFF techniques will allow control over localized pore morphologies and porosities to suit the requirements of different cell types within the same scaffold volume. This is achieved by incorporating different controllable macroscopic and microscopic design features on different regions of the same scaffold. Depending on the TE strategy used, this can be realized either using negative anisotropic scaffolds, positive anisotropic scaffolds, or a combination of both. Negative anisotropic scaffolds, often referred to as “mirror imaged” scaffolds, are fabricated based on guidance or entubulization TE strategies. Newly formed tissues growing into the scaffold will be guided and subsequentially differentiate directly into the anisotropic organization of the native tissues (indirect) [72,80,82,83,88,103]. On the other hand, positive anisotropic constructs are fabricated directly into the anisotropic organization of the native tissue, resembling the hierarchical structure of multiple cell types, and/or ECM compounds, such as collagen and elastin [34,45,96,134]. Scaffolds consisting of aligned fibers were found to possess controllable anisotropic mechanical properties and to dictate cellular morphology, with cell polarity following the prevailing fiber direction [125]. Under conditions of maximal fiber alignment, meniscal chondrocytes attached to these scaffolds and their directionality was prescribed in both the short and long term culture. It has previously been shown in organized monolayer cultures that fibroblasts deposit an organized ECM according to cell orientation [135], and that ligament fibroblasts on an aligned fiber meshes produce more collagen than on random meshes [94].

A common problem encountered when using conventional scaffolds for tissue engineering is the rapid formation of tissue on the outer edge, leading to the development of a necrotic core due to limitations of cell penetration and nutrient exchange [102,136,137]. Most tissues possess a network of blood and capillary vessels that perform this function in vivo, but engineering such a complex construct in vitro has been shown to be quite challenging [138,139]. Even avascular tissue, such as cartilage, has been shown challenging to engineer since the *in vivo* nutrient supply still cannot be adequately simulated in vitro [136]. A common approach used to overcome this issue is to utilize sophisticated culture systems or bioreactors to perfuse culture media around and/or through the scaffold [140,141,142]. Although these bioreactors have been successful, even in an optimized in vitro culture system, there is still a need to ensure tissue growth occurs evenly throughout the construct. Furthermore, for strategies where the scaffold alone will be implanted and the body is used as bioreactor, it is important to ensure that native tissue can infiltrate the whole scaffold to ensure adequate integration of the construct [143]. Another strategy to encourage tissue formation and cell differentiation within scaffolds, in vitro or in vivo, is to incorporate biological factors, such as growth factors, ECM compounds, or pharmaceutical agents which can also act as chemotactic factors to encourage cell migration and differentiation [144].

Patient specific data and scaffold structural properties required for regenerating specific tissues can be incorporated into scaffold design via CAD, often referred to as ‘reverse engineering’ [22]. SFF techniques can be easily automated and integrated with these imaging techniques to produce scaffolds that are customized in size and shape allowing tissue engineered grafts to be tailored for specific applications or even for individual patients [145]. In musculoskeletal applications, so far, these patient specific scaffolds have been used for the reconstruction of cranial [146,147], calvarial [148], and maxillofacial defects [149].

A key factor used to enhance the versatility of scaffold fabrication by SFF is construction of a scaffold matrix using a wide variety of biomaterials [145]. One emerging method is to fabricate a negative mold based on the scaffold design and cast the scaffold using desired polymeric and/or ceramic biomaterials. This alternative technique is known as ‘indirect’ SFF. Based on a lost mold technique by combining epoxy resin molds made by stereolithography and CAD data HA scaffolds with interconnecting pores have been created [150]. Despite a few fabrication inaccuracies, *in-vivo* experiments demonstrated osteoconductivity and biocompatibility in a minipig model. In addition, a series of biomimetic scaffolds have been fabricated by mold removal in combination with conventional sponge fabrication [151]. The fabricated scaffolds had interconnected pores ranging from 500–800 µm as specified by the prefabricated mold, and when local pores were formed they ranged from 10–300 µm depending on the local pore creating method. Similarly, an indirect SFF method for ceramic scaffolds with a defined and reproducible 3D porous architecture has been developed [152]. They reported ectopic bone formation for all scaffold and cell constructs. Manufacturing of collagen based scaffolds by using the SFF technique to fabricate a mold has also been reported [153].

The main restriction on casting with the conventional techniques was the inability to create molds to produce complex geometries and internal architecture but with indirect SFF conventional casting processes with these molds can meet specific tissue engineering requirements [145]. Indirect SFF allows use of a wider range of biomaterials or combinations of materials, such as composites and/or copolymers. However, some drawbacks still exist with this technology, including the use of organic solvents and the resolution of the SFF method. For example, the cast model copies errors and defects from the mold, such as cracks and dimensional changes. In addition, a method has to be developed to remove the mold precisely while preserving the cast scaffold intact without compromising its properties [145].

## 6. Conclusions

Although many options are available to fabricate anisotropic scaffolds for musculoskeletal tissue engineering, each method has its own set of strengths and weaknesses. For all techniques described, the choice of scaffold material seems to be endless since almost every polymer, natural or synthetic, can be used in combination with the desired technique. However, it has to be ensured that choice of materials for the scaffold is compatible with the selected method. Important considerations that should be made during the selection of materials used for musculoskeletal tissue engineering include mechanical properties of the material, scaffold design, degradation profile, bioactivity and biodegradability, as well as issues of cell seeding and vascularization.
